# The Behavioral Problems in 2.5–5 Years Old Children Linked with Former Neonatal/Infantile Surgical Parameters

**DOI:** 10.3390/children8050423

**Published:** 2021-05-20

**Authors:** Danguolė Rugytė, Giedrė Širvinskienė, Rima Kregždytė

**Affiliations:** 1 Department of Anesthesiology, Lithuanian University of Health Sciences, 44307 Kaunas, Lithuania; 2 Department of Health Psychology, Lithuanian University of Health Sciences, 44307 Kaunas, Lithuania; giedre.sirvinskiene@lsmuni.lt; 3 Health Research Institute, Lithuanian University of Health Sciences, 44307 Kaunas, Lithuania; 4 Department of Preventive Medicine, Lithuanian University of Health Sciences, 44307 Kaunas, Lithuania; rima.kregzdyte@lsmuni.lt; 5 Neuroscience Institute, Lithuanian University of Health Sciences, 44307 Kaunas, Lithuania

**Keywords:** surgery, neonates, infants, child behavior checklist, internalizing problems, externalizing problems, cerebral tissue oxygenation, near infrared spectroscopy

## Abstract

Studies report the link between exposure to major neonatal surgery and the risk of later neurodevelopmental disorders. The aim of this study was to find out the behavioral problem scores of 2.5–5 years old children who had undergone median/major non-cardiac surgery before the age of 90 days, and to relate these to intraoperative cerebral tissue oxygenation values (rSO_2_), perioperative duration of mechanical ventilation (DMV) and doses of sedative/analgesic agents. Internalizing (IP) and externalizing problems (EP) of 34 children were assessed using the CBCL for ages 1½–5. Median (range) IP and EP scores were 8.5 (2–42) and 15.5 (5–33), respectively and did not correlate with intraoperative rSO_2_. DMV correlated and was predictive for EP (β (95% CI) 0.095 (0.043; 0.148)). An aggregate variable “opioid dose per days of ventilation” was predictive for EP after adjusting for patients’ gestational age and age at the day of psychological assessment, after further adjustment for age at the day of surgery and for cumulative dose of benzodiazepines (β (95% CI 0.009 (0.003; 0.014) and 0.008 (0.002; 0.014), respectively). Neonatal/infantile intraoperative cerebral oxygenation was not associated with later behavioral problems. The risk factors for externalizing problems appeared to be similar to the risk factors in preterm infant population.

## 1. Introduction

Long-term developmental outcomes following major non-cardiac surgery for severe congenital anomalies or diseases in neonates and infants of the first several months of age are significantly affected [1,2]. Potential reasons underlying worse long-term effects are not clearly understood. Experimental animal models have demonstrated that rapid neuronal growth and synaptogenesis can be affected by anesthetics and sedative agents. In humans, extensive neuronal growth and synaptogenesis occur from late gestation to 12–24 months of age. This may predispose neonates and infants to the greatest risk for neurocognitive effects of sedatives, analgesics, and anesthetics used during perioperative period [3]. So far, though, despite the evidence of anesthetic toxicity in animals, no evidence exists for anesthesia related toxicity in humans.

Several other important potential mechanisms for impaired neurodevelopmental outcome after neonatal and early infantile surgery have been proposed. Cerebral white matter lesions detected by magnetic resonance imaging as well as cerebral abnormalities spotted by ultrasound during neonatal perioperative period have been demonstrated [4]. Perioperative electroencephalogram (EEG) changes and seizures were also described in post-surgical neonates [5]. Cerebral hypoxia, hypo- and hyper-perfusion, systemic hypo-tension or hypo/hypercapnia were proposed as the possible causes, outlining the importance of circulation and oxygenation/ventilation related factors and the need for better knowledge of cerebral autoregulatory capacity in term and preterm severely ill neonates.

With the greater appreciation of potential risk factors, attempts have been made to evaluate brain function during perioperative period and intensive care. Observation of cerebral tissue oxygenation by means of near infrared spectroscopy (NIRS) is becoming a bed-side monitoring tool along with other recent central nervous system monitoring modalities, such as EEG or dopplerography. It has been shown previously that cerebral tissue oxygenation may decrease during neonatal and infantile non-cardiac surgery and anesthesia [6,7]. So far, though, the association between altered cerebral oxygenation and long-term neurodevelopmental outcome has not been demonstrated [8].

Many neurodevelopmental tests have been used for long-term assessment of children following surgery [9]. However, up to now there is no uniform agreement as to what assessment tools are best to identify potential long-term effects associated with previous perioperative period.

The Child Behavior Checklist (CBCL) includes multidimensional behavioral assessment and covers the DSM diagnostic category-related scales. As early childhood is often an onset of behavioral problems which can reasonably be considered a high-risk factor for later mental health disorders, CBCL for ages 1.5–5 years (CBCL/1½–5) is widely used across clinical, educational, and research settings [10,11]. More is known about such risk factors of behavioral problems as socio-economic status, parental education, age, parental substance abuse, psychopathology, disciplinary practices, parental interaction, child sex, temperament [12], and early life stress (maltreatment, neglect, parental stress, family conflict etc.) [13]. However, understanding is still lacking on the impact of medical conditions on later behavioral child problems.

Along with other tools for developmental evaluation, behavioral assessment by means of CBCL has been described in previous reports on long-term effects of surgery and perioperative period. The results of these studies, though, are inconsistent. An earlier study by Ing et al. did not find differences in behavioral outcomes at the age of 10 years in children who had undergone surgery before the age of 3 years [14]. However, an increase in certain aspects of problematic behavior was found in a large recent epidemiological study, examining the association of number of surgeries and long-term outcome [15]. This study reports that exposure to surgery, especially multiple surgeries, was associated with more behavioral problems at ages between 8–12 and 15–19 years. A study which focused on the effects of the time of first anesthetic exposure revealed a trend towards increased risk of more disturbed behavioral development in children operated on at younger age, especially at the age of 0–6 months [16].

A recent review focusing on long-term outcome following surgery for congenital heart disease in infants before the age of 9 weeks reported an inconsistently increased risk for different aspects of behavioral problems in later childhood [17]. Hypoxemia, among the other perioperative factors, was most often reported to be associated with impaired behavioral and other developmental domains in the great majority of studies included in the review. Data relating long-term behavioral outcome with former perioperative variables, including cerebral oxygenation, following neonatal/infantile general surgery are missing. Therefore, the primary aim of the present study was to ascertain behavioral problem scores of preschool children who had undergone median/major non-cardiac surgery before the age of 90 days, and to relate these to intraoperative cerebral tissue oxygenation values. The secondary aim was to identify other possible perioperative predictors for increase in problem scores.

## 2. Materials and Methods

This prospective follow-up study was approved by Kaunas Regional Biomedical Research Ethics Committee (No. P1-BE-243/2012). Parents/legal guardians of included patients gave informed consent before participation in accordance with the Declaration of Helsinki. The study involved children who had undergone median/major non-cardiac surgery and had participated in a former clinical trial on intraoperative cerebral tissue oxygenation by means of NIRS (NCT02423369 at clinicaltrials.gov).

### 2.1. Previous Surgery Related Parameters Including Monitoring of Cerebral Oxygenation

Children, born term and preterm, had been operated on for abdominal, thoracic or genitourinary congenital malformations or diseases before the age of 90 days. During surgery anesthesia was maintained with inhalational anesthetic (sevoflurane), opioid and muscle relaxant. Mechanical lung ventilation was used in all infants. Patients who had undergone neurosurgical, ear, nose, throat and eye surgery, had bronchopulmonary dysplasia, esophageal atresia or tracheo-esophageal fistula, diaphragmatic hernia, malignancies, or were American Society of Anesthesiology physical status (ASA) class 5 were not included.

Before induction of anesthesia, two pediatric cerebral sensors were placed bilaterally to the forehead region, and cerebral oxygenation (rSO_2_) monitoring (INVOS^®^, SOMANETICS (Medtronic, Minneapolis, MN, USA)) was started and continuously applied throughout surgery until the wound closure. Data were captured with a sampling interval of 5 s and recorded every 5 min. The difference between maximal and minimal intraoperative rSO_2_ value (maximal intraoperative rSO_2_ change) and mean intraoperative rSO_2_ value were calculated for every patient. More methodological details can be found elsewhere [7].

Data on the perioperative duration of mechanical lung ventilation (DMV) and the use of opioids and benzodiazepines covering the period from 24 h prior to 72 h after surgery were collected. Cumulative benzodiazepine dose was calculated (µg kg^−1^) for each patient, assuming benzodiazepine conversion ratio diazepam:midazolam = 1:1 [18]. Cumulative dose of perioperative opioids was calculated, assuming opioid conversion ratio 10 µg fentanyl = 1 mg morphine [19]. Cumulative opioid dose was expressed in fentanyl equivalents (µg kg^−1^).

### 2.2. Psychological Evaluation

At the age of 2.5–5 years, behavior of children was assessed using the Child Behavior Checklist for Ages 1½–5 (CBCL/1½–5) [10], filled in by the parents. The Lithuanian version of the tool was used, which was adapted and standardized in Lithuania [20]. The CBCL/1½–5 questionnaire consists of 99 items describing various manifestations of young children’s behavior. The statements in the questionnaire cover three aspects of a broad spectrum of problems: internalizing, externalizing, and total problems. The scale of internalizing problems (IP) consists of emotionality, anxiety/depressiveness, somatic complaints, and withdrawal subscales. The scale of externalizing problems (EP) includes attention problems and aggressive behavior subscales. Total problems (TP) scale comprises internalizing and externalizing problem scales, sleep problems subscale, and other statements. Parents provided information on different statements and rated the child behavior using a 3-point scale (0—not true, 1—somewhat or sometimes true, 2—very true or often true) based on behaviors in the past 2 months. Raw scores for each scale were calculated and used for statistical analysis. The comparative analysis in the sample of children aged 2.5–5 years was possible, as the same norms are specified for the age span 1½–5 years using CBCL/1½–5 [10].

The questionnaires were distributed and returned by mail. Socio-demographic characteristics were also collected.

### 2.3. Outcome Measures

The primary outcome measures were CBCL/1½–5 scores (TP, IP, EP) and their relationship with mean intraoperative rSO_2_ and maximal intraoperative rSO_2_ change. The secondary outcome measures were the relationship of TP, IP and EP with perioperative DMV and cumulative doses of sedative agents. The relationship with other potential variables was also explored and included in the analysis.

### 2.4. Statistical Analysis

Shapiro-Wilk test was used to assess the normality of distribution of continuous data. Primary outcome measures, i.e., CBCL/1½–5 scores: TP, EP, but not IP, followed normal distribution. Distribution of some demographic and clinical variables was not normal. Thus, we used mean and median (range) for the consistency of presentation of demographic and clinical data. Categorical and binomial variables are presented as number of patients (%). Mann-Whitney U test was used to compare TP, EP, IP scores, mean intraoperative rSO_2_ values, maximal intraoperative rSO_2_ change, perioperative DMV and cumulative doses of sedative agents between term and preterm patients.

Spearman rank correlation was used to assess associations of problem scale scores and mean intraoperative rSO_2_ values, maximal intraoperative rSO_2_ change, perioperative DMV and the cumulative doses of sedative agents.

The predictive value for the problem scale scores (TP and EP) including DMV and the doses of perioperative sedative agents adjusted for relevant cofactors was assessed using univariate and multiple linear regression analysis [21]. Regression coefficients (β) and their 95% confidence intervals (CI) were calculated; *p* < 0.05 was considered statistically significant.

To find out the number of children to be included in future studies in order to demonstrate the predictive value of intraoperative cerebral oxygenation for total problem scale score, power analysis based on expected parameters in multiple linear regression analysis, such as effect size and number of predictors, given the significance level and power, was performed using G*Power software.

## 3. Results

**Figure 1:**
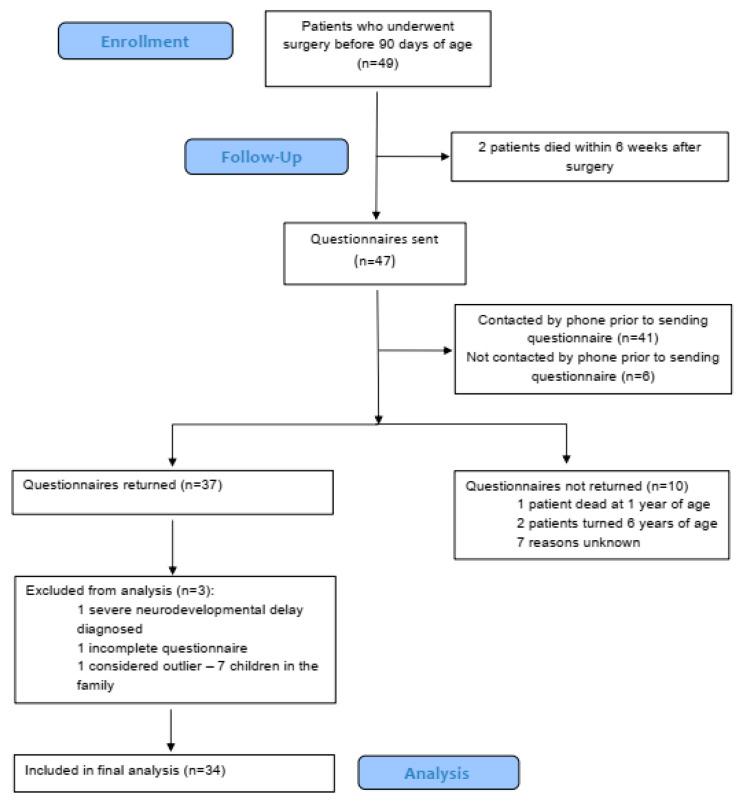
Flowchart of patients included in the psychological assessment.

The flowchart of patients included in psychological assessment is shown in Figure 1. Questionnaires were sent to the parents of 47 children, 37 questionnaires were returned, giving a response rate of 78.7%, and data of 34 patients were analyzed (Figure 1).

### 3.1. Socio-Demographic and Clinical Characteristics and Problem Scale Scores of the Studied Patients

Socio-demographic characteristics, CBCL/1½–5 scores, and clinical characteristics during previous surgery of the studied patients are shown in Table 1 (socio-demographic and clinical characteristics of 10 ‘lost to follow-up’ patients are shown in Table S1). Mean age at psychological assessment was 4.3 years and ranged from 2.8 to 5.9. Gender distribution was almost equal, and problem scale scores were within the reported range of the international population [22]. The great majority of patients had undergone surgery for major gastrointestinal, urogenital and abdominal wall diseases or abnormalities, with a mean duration of 1.5 h.

There were 10 former preterm patients, of whom six were still preterm at the time of surgery. There were no differences between term and preterm patients in TP, EP or IP, intraoperative cerebral rSO_2_ values and cumulative perioperative doses of benzodiazepines (Table 2). DMV and cumulative perioperative doses of opioids tended to be higher in preterm infants, however, without statistical significance (Table 2).

### 3.2. Associations between Previous Perioperative Parameters and Behavioral Problem Scores

Mean intraoperative rSO_2_ values and maximal intraoperative rSO_2_ change did not correlate with TP (Spearman’s rho 0.2016; *p* = 0.261 and −0.1783; *p* = 0.321, respectively), IP (Spearman’s rho 0.1897; *p* = 0.291 and −0.096; *p* = 0.595, respectively) or EP (Spearman’s rho 0.1171; *p* = 0.516 and −0.194; *p* = 0.279, respectively) and were not predictive for TP, IP or EP. Power analysis, based on our present data, showed that in order to obtain a predictive value of mean intraoperative cerebral oxygenation for TP scale score, assuming power of 0.9, two-tailed significance of 0.05 and including 5 possible confounding factors, at least 555 children should be included in future studies.

**Figure 2:**
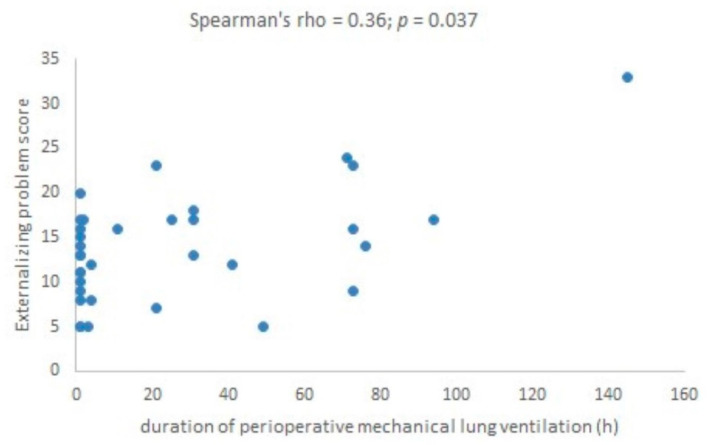
Correlation between the duration of perioperative mechanical lung ventilation and externalizing problem scale scores.

DMV correlated with EP (Spearman’s rho = 0.36; *p* = 0.037) (Figure 2), but not with TP and IP scores (Spearman’s rho = 0.15; *p* = 0.404 and Spearman’s rho = −0.03; *p* = 0.873, respectively).

Patients’ age at the day of psychological assessment negatively correlated with EP scores (Spearman’s rho = −0.398, *p* = 0.0198).

DMV was positively predictive for EP when adjusted for patients’ age at the day of psychological assessment and gestational age by multiple linear regression (Table 3)

**Figure 3:**
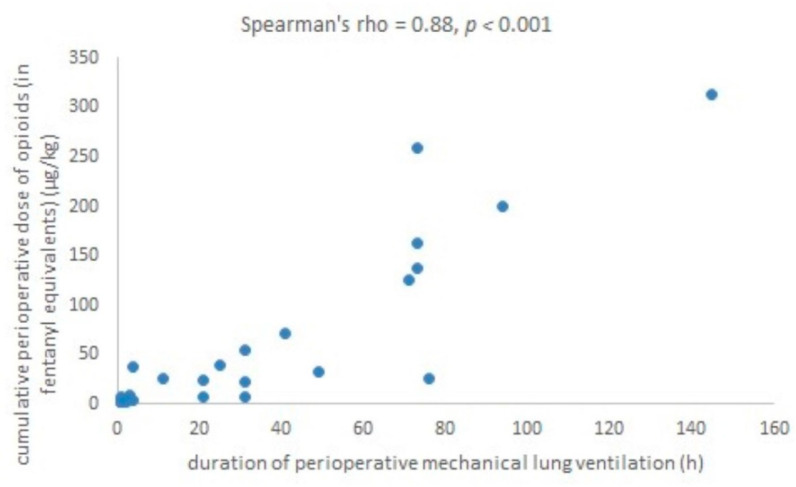
Correlation between duration of perioperative mechanical ventilation and cumulative perioperative dose of opioids.

The cumulative doses of opioids and sedative agents did not correlate with TP (Spearman’s rho = 0.11, *p* = 0.530 and Spearman’s rho = 0.14, *p* = 0.431, respectively), EP (Spearman’s rho = 0.26, *p* = 0.141 and Spearman’s rho = 0.22, *p* = 0.204, respectively) and IP scores (Spearman’s rho = −0.04, *p* = 0.843 and Spearman’s rho = 0.11, *p* = 0.553, respectively). However, the cumulative opioid dose strongly correlated with DMV (Spearman’s rho = 0.88, *p* = 0.000) (Figure 3). Thus, the variable “opioid dose per days of ventilation” (ODDV) was created ((opioid dose +1) × (ventilation h + 1)/24). ODDV was positively predictive for EP after adjustment for patients’ gestational age and age at the day of psychological assessment, as well as after further adjustment for age at the day of surgery and for cumulative dose of benzodiazepines (Table 3). Full multiple regression models including confounding variables are shown in Tables S2–S6.

Neither length of stay in the intensive care unit nor the total number of surgeries up to psychological assessment, nor social variables, such as family status, maternal education and total number of children (up till 16 years) in the family, correlated with the problem scale scores.

## 4. Discussion

The main outcome of this study was behavioral problem scale scores at the age of 1.5–5 years and their association with cerebral tissue oxygenation values by means of NIRS during former neonatal/infantile surgery.

Externalizing, internalizing and total problem scales scores found in the present study were within the range of the international population. A multicultural study including 24 countries (including Lithuania) from around the world and almost 20,000 parent reports found the omni-cultural mean for total problem scores of 33.3 (range 17.2–47.5). Omni-cultural mean (range) for internalizing problems was reported to be 9.6 (3.9–13.9) and, for externalizing problems, 12.0 (6.7–16.9). Comparison with our country’s general population scores may be preferable; however, the authors point out small to medium differences across countries and more variance within than between countries, making comparison with the omni-cultural mean reasonable [22]. Thus, it seems that a cohort of our patients, who had undergone median/major neonatal/infantile surgery up till 3 months of age for diverse abdominal, urogenital and other congenital non-cardiac abnormalities, has similar long-term behavioral outcome at mean 4.3 years of age as an omni-cultural general population. It is worth mentioning, though, that our studied patient cohort did not include children who had suffered such severe pathologies as esophageal atresia or diaphragmatic hernia, which had been associated with worse outcome in certain neurodevelopmental domains in previous studies [23,24].

An association between long-term outcome and perioperative cerebral oxygenation was demonstrated in children after cardiac surgery [25]. However, no study describes the relationship of intraoperative cerebral oxygenation and long-term outcome following neonatal/infantile median/major general surgery. As neonates are at significantly higher risk for perioperative complications and worse outcome compared to older children [3], attempts are made to minimize this risk and to find out predictive markers of potential damage. A study by Verhagen et al. found a relationship between abnormalities of cerebral tissue oxygenation during the first 2 weeks of life and several neurodevelopmental outcome domains, but no relationship with CBCL at age 2–3 years in preterm children [26]. Their finding, though, was not confirmed by a recent large multicenter study that found no evidence of prognostic value of cerebral tissue oxygenation in a cohort of extremely preterm neonates [27]. In the latter study, however, monitoring was limited only to the first three days of life. Similarly, no predictive value for later cognitive and motor developmental disorders of cerebral tissue oxygenation was found in a cohort of preterm infants with sepsis, although, oxygenation values remained within the considered normal range [28].

The clinical value of intraoperative cerebral oxygenation monitoring on short-term behavior 7 days after general surgery in 2–12 year old children was demonstrated in a recent study [29]. Negative postoperative behavior assessed using the Post-Hospital Behavior Questionnaire was associated with an intraoperative decrease from baseline of cerebral oxygenation values. Long-term effects, though, remain unclear.

In the present study we found no relationship between behavioral problem scores at the mean age of 4.3 years and cerebral oxygenation values during former neonatal/infantile surgery. However, short duration of oxygenation monitoring and small number of included patients prevent drawing firm conclusions. Based on the results of the present study, we calculated that over 500 patients need to be included in future studies in order to demonstrate any predictive value of intraoperative cerebral oxygenation for increase in long-term behavioral problems.

Nevertheless, the externalizing problem score was associated with the duration of perioperative mechanical lung ventilation, which tended to be longer in preterm infants in the present study. This association is in line with previous reports, where every day of mechanical ventilation was significantly associated with neurodevelopmental and behavioral outcome at the age of 2 years in former preterm infants [30]. Furthermore, as mechanical ventilation is usually accompanied by the use of sedatives or opioids, when combining ventilation hours and opioid dose (ODDV), we found even stronger association with externalizing problem scores. Opioid—morphine dose in mechanically ventilated preterm neonates was shown to be positively associated with more behavioral problems at later age [31], and the recent study has related behavioral outcome to genes associated with morphine metabolism, showing that some infants may be potentially more prone to develop negative behavioral problems than others [32].

The relationship between externalizing problem scores and ODDV remained significant after adjustment for gestational age, age at the day of surgery and perioperative dose of benzodiazepines. Prematurity as well as preterm age at the day of surgery were associated with worse neurodevelopmental and behavioral outcome in several previous studies [33,34,35]. Benzodiazepines were also shown to be related with neuronal damage in experimental animal models [3] as well as with worse long-term outcome in humans [36]. Therefore, these factors were included as confounders in the multiple regression model in the present study.

We further included child’s age at psychological assessment as a confounder as we found negative correlation between externalizing problems and child’s age. International comparisons of internalizing and externalizing problems across 24 societies revealed that, for externalizing problems, boys and younger children scored higher than girls and older children; however gender and age differences were all very small (effect sizes < 1%) [22].

### Limitations

We used a single tool for long-term developmental assessment. So far, no consensus exists on the ideal assessment tools to investigate long-term effects of former perioperative period/s, while CBCL was used to assess the long-lasting effects of potential risk factors in former preterm neonates and in children who had undergone early surgery [16,31,32,37]. Stratification by age is frequent in behavioral outcome studies, including CBCL. Our studied sample included an age span from 33 to 71 months (2.8–5.9 years). According to the original study by Achenbach and Rescorla on CBCL/1½–5 in a large population of 18 to 71 months old children, only minimal gender and age differences exist [10]. In accordance with finding by Achenbach and Rescorla, a large Danish study that assessed 1½, 2, 3, 4 and 5 years old children has shown that, with regard to total, internalizing and externalizing raw scale scores, children aged 3 to 5 years can be assessed as a single group [38].

The response rate to CBCL questionnaire was 78.7%, which is comparable to other reports [14]. Although clinical variables of interest in infants who were lost to follow up did not significantly differ from the studied group, lost-to follow-up had negative effect on our sample size. Adequate sample size in multiple linear regression models is a question of debate and may depend on the number of included independent variables (predictors). Earlier studies suggested at least 10 to 20 cases per independent variable in multiple linear regression models [39]. However, Austin and Steyerberg in 2015 demonstrated that the number of subjects (cases) per independent variable, ranging from just 2 to 50, results in less than 10% variation of the β regression coefficient and reliably represents the studied sample [21]. This sample, though, must adequately represent the specific population intended to be studied [40]. We did not include children who had surgeries for esophageal atresia, congenital diaphragmatic hernia, or ear, throat, nose abnormalities, conditions that had been associated with worse long-term outcome previously [23,24,41]. Thus, our studied patients had surgeries for anomalies or diseases that do not directly interfere with respiratory, cardiovascular or central nervous system, and abdominal or urological malformations were predominant. Ten preterm children were also included in our study, therefore adjustment for gestational age was made when analyzing the results. Duration of monitoring of cerebral tissue oxygenation, exceptional during surgical procedure, was short compared to the whole perioperative period, which may cover over 24 h following major surgery. Thus, a considerable amount of information may be missing. Finally, pain assessment was not included in this study, though pain itself was shown to have long-lasting effects on behavior [31,37]. Epidemiological studies also showed that number of surgeries in early childhood may be associated with later long-term outcome [15,35]. Although there is no sufficient evidence to confirm this relationship, we cannot exclude that multiple surgeries before psychological assessment in some of our studied patients influenced behavioral outcome in the present study.

## 5. Conclusions

Behavioral problem scores assessed by CBCL/1½–5 at the mean age of 4.3 years in children who had undergone median/major non-cardiac surgery before 3 months of age were within the range of reported scores of a general international population. In addition, no relationship was found between problem scores and cerebral tissue oxygenation during former neonatal/infantile surgery. The risk factors for externalizing problems appeared to be similar to the risk factors in the preterm infant population, showing the relationship with the duration of perioperative mechanical ventilation and the dose of opioids. The results of this study, though, may be influenced by a small number of patients and confounding factors, which should be considered when planning forthcoming investigations. Much larger and even procedure specific studies with a broad spectrum of neurodevelopmental assessment tools may provide more comprehensive information in future.

## Figures and Tables

**Table 1:** Socio-demographic characteristics, problem scale scores assessed by Child Behavior Checklist for Ages 1½–5 (CBCL/1½–5) and clinical characteristics of studied patients during previous surgery up till age of 90 days (*n* = 34). Values are mean; median (range) or number of patients (%).

Demographic, Social Characteristics and Problem Scale Scores	
Age (months) at psychological assessment	52.8; 52 (33–71)
33–35	2 (5.9%)
36–60	24 (70.6%)
61–71	8 (23.5%)
Male gender	18 (52.94%)
Total number of surgeries up till behavioral assessment	2.6; 2 (1–18)
Total number of children (up till 16 years) in the family	2; 2 (1–6)
Family status:	
Registered marriage/Cohabitation	28 (82.35%)
Single	4 (11.76%)
Not reported	2 (5.89%)
Maternal education:	
Secondary or lower	13 (38.24%)
Higher	21 (61.76%)
Problem scale scores:	
Total	32.4; 31.5 (10–82)
Externalizing	13.9; 15.5 (5–33)
Internalizing	10.1; 8.5 (2–42)
Clinical characteristics during previous surgery up till age of 90 days	
Gestational age (weeks)	38; 38.5 (30–41)
Preterm:	10 (29.41%)
Gestation 30–33 weeks	1
Gestation 34–36	8
Gestation 37 weeks	1
Age at the day of surgery (days)	17.1; 8.5 (0–90)
Preterm at the day of surgery	6 (17.65%)
Weight at the day of surgery (g)	3372.6; 3400 (1800–5800)
Surgery:	
Gastrointestinal (including anorectal)	17 (50%)
Abdominal wall defects	6 (17.65%)
Urogenital	6 (17.65%)
Teratoma	3 (8.82%)
Thoracic	1 (2.94%)
Biliary	1 (2.94%)
ASA class:	
2	19 (55.88%)
3	13 (38.24%)
4	2 (5.88%)
Duration of anesthesia (min)	87.5; 80 (30–195)
Mean intraoperative cerebral rSO_2_ value (%)	79.5; 79.9 (54.1–93.6)
Maximal intraoperative rSO_2_ change	17.5; 16.5 (5.0–35.5)
Perioperative ventilation hours	25.3; 3 (0–144)
Cumulative perioperative dose of opioids (µg kg^−1^) ^1^	47.3; 7.3 (1.2–312.8)
Cumulative perioperative dose of benzodiazepines (µg kg^−1^) ^2^	395; 275 (0–1840)
Length of stay in the intensive care unit (days)	4.8; 2.5 (0–22)

^1^ Cumulative dose was calculated, assuming opioid conversion ratio 1 mg morphine = 10 µg fentanyl and expressed in fentanyl equivalents (µg kg^−1^); ^2^ Cumulative benzodiazepine dose (diazepam and midazolam) assumes a ratio 1:1 in µg kg^−1^.

**Table 2:** Comparison of primary and secondary outcome variables between term and preterm patients. Values are mean; median (range).

Variable	Term Patients (*n* = 24)	Preterm Patients (*n* = 10)
Total behavioral problem scale scores	32.5; 33 (10–82)	32.2; 28.5 (11–57)
Externalizing problem scale scores	13.0; 13 (5–23)	16; 15 (5–33)
Internalizing problem scale scores	10.8; 9.5 (2–42)	8.5; 8 (3–17)
Mean intraoperative cerebral rSO_2_ value (%)	79.9; 79.7 (56.9–92.7)	78.6; 83.6 (54.1–93.6)
Maximal intraoperative rSO_2_ change	16.8; 15.8 (6–35.5)	19.2; 19 (5–32.5)
Perioperative ventilation hours	16.3; 2.5 (0–75)	46.7; 44 (0–144) ^1^
Cumulative perioperative dose of opioids (µg kg^−1^) ^2^	24.9; 7.1 (2–163)	101.1; 51.4 (1.2–312.8) ^3^
Cumulative perioperative dose of benzodiazepines (µg kg^−1^) ^4^	388.8; 275 (0–1840)	410; 250 (0–1500)

^1^*p* = 0.196, compared to term patients; ^2^ Cumulative dose was calculated, assuming opioid conversion ratio 1 mg morphine = 10 µg fentanyl and expressed in fentanyl equivalents (µg kg^−1^); ^3^ *p* = 0.290, compared to term patients; ^4^ Cumulative benzodiazepine dose (diazepam and midazolam) assumes a ratio 1:1 in µg kg^−1^.

**Table 3:** Predictive value of perioperative mechanical lung ventilation (DMV) hours and perioperative opioid dose per days of ventilation (ODDV) for externalizing problem (EP) score by linear regression models.

	Regression Beta Coefficient (β)	95% Confidence Interval	*p* Value
Perioperative ventilation hours (DMV) ^1^			
	0.095	0.043; 0.148	0.001
DMV ^2^	0.080	0.011; 0.148	0.025
DMV ^3^	0.066	−0.009; 0.140	0.083
opioid dose per days of ventilation (ODDV) ^1^			
	0.010	0.005; 0.015	<0.001
ODDV ^2^	0.009	0.003; 0.014	0.003
ODDV ^3^	0.008	0.002; 0.013	0.007
ODDV ^4^	0.008	0.002; 0.014	0.011

^1^ univariate linear regression; ^2^ multiple linear regression β adjusted for patient’s age at the day of psychological assessment and gestational age; ^3^ multiple linear regression β adjusted for patient’s age at the day of psychological assessment, gestational age and age at the day of surgery; ^4^ multiple linear regression β adjusted for patient’s age at the day of psychological assessment, gestational age, age at the day of surgery and cumulative perioperative dose of benzodiazepines.

## Supplementary Materials

The following are available online at https://www.mdpi.com/article/10.3390/children8050423/s1, Table S1: Socio-demographic and clinical characteristics of the lost to follow-up patients (*n* = 10), Table S2: Multiple linear regression model showing the association of perioperative mechanical lung ventilation (DMV) hours with externalizing problem score adjusted for two confounding variables, Table S3: Multiple linear regression model showing the association of perioperative mechanical lung ventilation (DMV) hours with externalizing problem score adjusted for three confounding variables, Table S4: Multiple linear regression model showing the association of perioperative opioid dose per days of ventilation (ODDV) with externalizing problem score adjusted for two confounding variables, Table S5: Multiple linear regression model showing the association of perioperative opioid dose per days of ventilation (ODDV) with externalizing problem score adjusted for three confounding variables, Table S6: Multiple linear regression model showing the association of perioperative opioid dose per days of ventilation (ODDV) with externalizing problem score adjusted for four confounding variables.
