# Supplementary material for: The Behavioral Problems in 2.5–5 Years Old Children Linked with Former Neonatal/Infantile Surgical Parameters
Source: Children (Basel). 2021 May 20;8(5):423. doi: 10.3390/children8050423 (PMC8160720; doi:10.3390/children8050423)
Supplement: Supplementary file 1 [file children-08-00423-s001.zip › children-1199270-supplementary.pdf]

Table S1. Socio-demographic and clinical characteristics of the lost to follow-up patients ($n = 10$). Values are mean; median (range) or number of patients (%).

Gestational age (weeks)	38.4; 39.5 (33-40)
Preterm:	2 (20%)
Gestation 30-33 weeks	1
Gestation 34-36	1
Gestation 37 weeks	0
Age at the day of surgery (days)	34.4; 33 (2-90) [1]
Preterm at the day of surgery	0
Weight at the day of surgery (g)	4158.7; 3882.5 (3000-6300) [1]
Surgery:	
Gastrointestinal (including anorectal)	4 (40 %)
Abdominal wall defects	2 (20 %)
Urogenital	2 (20 %)
Teratoma	2 (20 %)
Thoracic	-
Biliary	-
ASA class:	
1	2 (20 %) [1]
2	6 (60 %)
3	2 (20 %)
4	-
Duration of anesthesia (min)	93; 70 (35-260)
Mean intraoperative cerebral rSO_2 value (%)	81.1; 82.1 (70.5-89)
Maximal intraoperative rSO_2 change	15.3; 14.5 (7-31)
Perioperative ventilation hours	14.2; 2.5 (1-61)
Cumulative perioperative dose of opioids ($\mu g\ kg^{-1}$)	29.48; 3.65 (1.1-222.6)
Cumulative perioperative dose of benzodiazepines ($\mu g\ kg^{-1}$)	340; 0 (0-1600)
Length of stay in the intensive care unit (days)	3.7; 2 (0-20)

$p < 0.05$, compared to the studied group (by Mann-Whitney and Chi-squared tests, where appropriate).

Table S2. Multiple linear regression model showing the association of perioperative mechanical lung ventilation (DMV) hours with externalizing problem score adjusted for 2 confounding variables (adjusted $R^2 = 0.242$; $p = 0.010$)

Variable	Regression beta coefficient (β)	95% confidence interval
DMV [1]	0.080	0.011; 0.148
Patient's age at the day of psychological assessment (months)	-0.08	-0.31; 0.16
gestational age (weeks)	-0.14	-0.95; 0.66

[1] $p = 0.025$

Table S3. Multiple linear regression model showing the association of perioperative mechanical lung ventilation (DMV) hours with externalizing problem score adjusted for 3 confounding variables (adjusted $R^2 = 0.241$; $p = 0.016$)

Variable	Regression beta coefficient (β)	95% confidence interval
DMV [1]	0.066	-0.009; 0.140
Patient's age at the day of psychological assessment (months)	-0.07	-0.31; 0.16
gestational age (weeks)	-0.34	-1.24; 0.57
age at the day of surgery (days)	-0.05	-0.15; 0.05

[1] $p = 0.083$

Table S4. Multiple linear regression model showing the association of perioperative opioid dose per days of ventilation (ODDV) with externalizing problem score adjusted for 2 confounding variables (adjusted $R^2 = 0.335$; $p = 0.002$)

Variable	Regression beta coefficient (β)	95% confidence interval
ODDV [1]	0.009	0.003; 0.014
Patient's age at the day of psychological assessment (months)	-0.12	-0.31; 0.07
gestational age (weeks)	-0.04	-0.80; 0.73

[1] $p = 0.003$

Table S5. Multiple linear regression model showing the association of perioperative opioid dose per days of ventilation (ODDV) with externalizing problem score adjusted for 3 confounding variables (adjusted $R^2 = 0.344$; $p = 0.002$)

Variable	Regression beta coefficient (β)	95% confidence interval
ODDV [1]	0.008	0.002; 0.013
Patient's age at the day of psychological assessment (months)	-0.096	-0.29; 0.099
gestational age (weeks)	-0.24	-1.07; 0.60
age at the day of surgery (days)	-0.05	-0.14; 0.04

[1] $p = 0.007$

Table S6. Multiple linear regression model showing the association of perioperative opioid dose per days of ventilation (ODDV) with externalizing problem score adjusted for 4 confounding variables (adjusted $R^2 = 0.321$; $p = 0.006$)

Variable	Regression beta coefficient (β)	95% confidence interval
ODDV [1]	0.008	0.002; 0.014
Patient's age at the day of psychological assessment (months)	-0.098	-0.30; 0.10
gestational age (weeks)	-0.23	-1.08; 0.62
age at the day of surgery (days)	-0.05	-0.15; 0.04
perioperative dose of benzodiazepines (μg kg^{-1})	-0.0002	-0.005; 0.004

[1] $p = 0.011$
